# RevManHAL: towards automatic text generation in systematic reviews

**DOI:** 10.1186/s13643-017-0421-y

**Published:** 2017-02-09

**Authors:** Mercedes Torres Torres, Clive E. Adams

**Affiliations:** 1 Computer Vision Lab, University of Nottingham, Nottingham, UK; 2 Cochrane Schizophrenia Group, Institute of Mental Health, University of Nottingham, Triumph Road, Nottingham, NG7 2TU UK

## Abstract

**Background:**

Systematic reviews are a key part of healthcare evaluation. They involve important painstaking but repetitive work. A major producer of systematic reviews, the Cochrane Collaboration, employs Review Manager (RevMan) programme—a software which assists reviewers and produces XML-structured files. This paper describes an add-on programme (RevManHAL) which helps auto-generate the abstract, results and discussion sections of RevMan-generated reviews in multiple languages. The paper also describes future developments for RevManHAL.

**Methods:**

RevManHAL was created in Java using NetBeans by a programmer working full time for 2 months.

**Results:**

The resulting open-source programme uses editable phrase banks to envelop text/numbers from within the prepared RevMan file in formatted readable text of a chosen language. In this way, considerable parts of the review’s ‘abstract’, ‘results’ and ‘discussion’ sections are created and a phrase added to ‘acknowledgements’.

**Conclusion:**

RevManHAL’s output needs to be checked by reviewers, but already, from our experience within the Cochrane Schizophrenia Group (200 maintained reviews, 900 reviewers), RevManHAL has saved much time which is better employed thinking about the meaning of the data rather than restating them. Many more functions will become possible as review writing becomes increasingly automated.

## Background

Producing systematic reviews in healthcare is a painstaking process of question setting, identification of relevant studies, data extraction, synthesis and, finally, write-up [1]. Often, because each stage necessitates different skills, systematic reviewing is a prolonged team effort. Many reviews take so long to produce that they are out of date before they are published [2].

Recognising the need for evidence to be current, the Cochrane Collaboration produces high-quality systematic reviews and attempts to maintain them [3]. Despite being an organisation focused on electronic publication [4] and having the infrastructure to approach the ideal of maintained up-to-date best evidence [5], production time is slow [6] and most reviews are considerably out of date [2].

Much effort has already been invested in machine-assisted production of healthcare systematic reviews. For over two decades, the Review Manager (RevMan) programme has automatically produced structures, tables, calculations and graphics from inputted data [7]. Teams have been investing in more automated data identification and extraction tools [8]. There is wide recognition of the need for up-to-date synthesis of data on effects of health treatments [9] but, at the same time, acknowledgement of the impossibility of relying solely on human effort [10]. A production line of software is forming. Some initiatives help auto-search for relevant papers [11], while others help sift out relevant studies, begin the process of data extraction from existing publications or undertake the synthesis and graphical display of data [8]. However, this production line does not function smoothly. It is not integrated enough and not formed by a necessary global collaboration. This paper describes one such programme towards the end of the poorly co-ordinated production line. It is a publicly available initiative which allows auto-generation of multilingual text within Cochrane’s open-access RevMan programme [7]. We recognise that this is proof-of-concept software and not universally compatible. However, at the same time, it is functional, links with a very commonly used package (RevMan) and saves–or at least redirects–countless hours of researcher time. For example, the ‘effects of interventions’ section of a Cochrane review often takes hours to transcribe data from data tables and to create and format the accompanying text. RevManHAL produces formatted text with accompanying numerical data in seconds. The necessary checking, thereafter, takes, approximately, an additional half hour. This letter is to alert users of RevMan to this additional tool and to raise awareness of how auto-generation of increasing proportions of text in multiple languages in systematic reviews is possible.

## RevMan

**Fig. 1:**
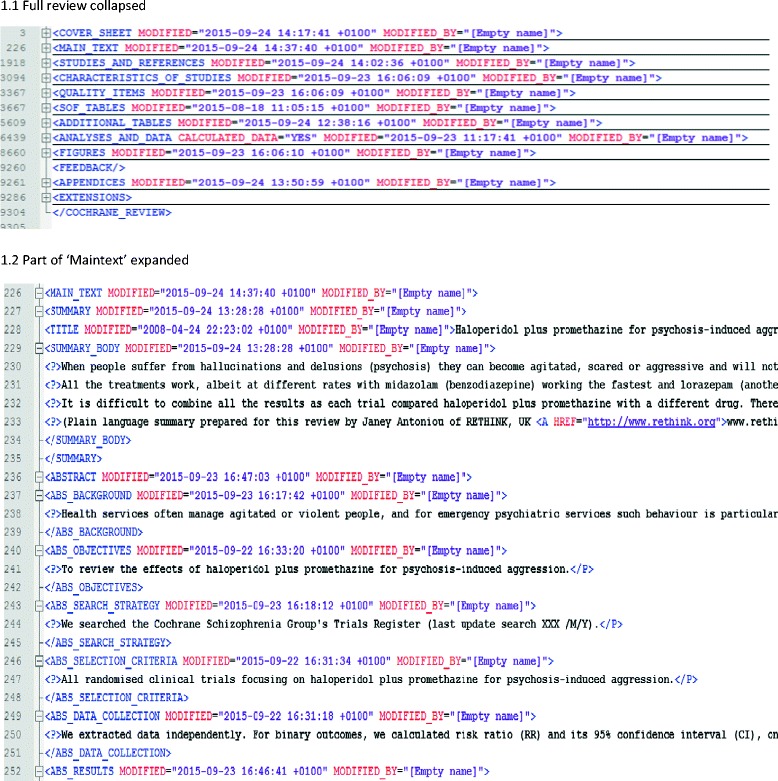
XML structure of a section of the RM5 file

RevMan is produced by the Informatics and Knowledge Management Department of the Cochrane Collaboration. It is an open-access text editor in which reviewers input quantitative and qualitative data related to their review into structured templates. The programme’s internal calculator allows limited synthesis and graphical presentation of data. RevMan outputs RM5 files, already marked up for electronic publication in various formats. In essence, RM5 files follow an XML format, with hierarchical sections encased between relevant tags (such as ‘abstract’ and ‘intervention’) (Fig. 1).

## Limitations of RevMan

While revolutionary in the 1990s it is now obvious that RevMan has important limitations. In particular, writing reviews within RevMan suffers from not facilitating repetition. Out of necessity, there is the need to repeat. One reader may be more comfortable with numbers and graphs, another may prefer text, and a third a combination. In some ways, RevMan is not repetitive enough. Most of the world do not read English as their first language, yet there is no functionality to help repeat the findings in other languages to assist wider dissemination. Currently, data entered into RevMan tables should, mostly, have some parallel text within the written ‘results’ if not the ‘abstract’ and ‘discussion’. Copying from the data input and calculation part of this widely used programme is still heavily manual and prone to mistakes. This slows the whole process down dampening the morale of reviewers and discouraging participation.

## RevManHAL

Data in RevMan are stored in structured format. This affords opportunities for automatic seeding of other sections of the review. RevManHAL, although currently not endorsed by the Cochrane Collaboration, is an open-access programme [12] created with the aim of solving some of the major issues reviewers encounter while producing documents in RevMan. (HAL was named in homage to the film 2001: A Space Odyssey in which a computer named HAL takes somewhat too much control [13].) In particular, RevManHAL aims to facilitate the laborious process of writing reviews by allowing users to create and edit text that can be automatically reused and inserted in all their reviews. This decreases the time that reviewers spend on major parts of the ‘results’, ‘discussion’ and ‘abstract’ sections of the reviews. This text can be in any language that reads left to right.

RevManHAL, v.4 and below, was created in Java using NetBeans, a popular programming language which benefits from being multi-platform.

By Version 4.2, RevManHAL offers two main functions:

1. Creating, editing and managing automatic text. Sections where HAL is able to input text are:
  - Abstract: using editable generic text stored by RevManHAL along with data from the Title and Summary of Findings (as long as it is in the simplest format) in RevMan. Each RevMan-generated review may contain one or two tables summarising the full review with a maximum of seven quality-rated outcomes, often, in turn, generated using the GRADEpro system [14]. These useful tables, if in their simplest form, can be employed by RevManHAL and the numerical data and quality rating seeded into the relevant parts of the review’s abstract. This information is bounded by generic and editable text making the end product readable. In addition, the structure of the title of the review (‘intervention’ for ‘problem’) facilitates production of generic text in sections of the abstract other than the results subsection.
  - Results of the search: using editable generic text stored by RevManHAL along with data from the PRISMA (Preferred Reporting Items for Systematic Reviews and Meta-Analyses) figure. Most reviews contain a figure describing the process of sifting the results of the search down to the final data set of studies (the PRISMA figure). These sources can be used to auto-generate text within the abstract, results and discussion sections.
  - Results—effects of interventions: using editable generic text stored by RevManHAL along with data from the RevMan files’ Analysis and Data table. The editable generic text within HAL may employ several ways of saying the same thing, and the machine then randomly chooses which to use—increasing the fluency and readability of the final product. For example, a finding could be statistically significant. This could be stated as ‘this finding was statistically significant’ or equally well as ‘for this outcome the difference reached conventional levels of statistical significance’ or ‘this result highlighted the clear difference between intervention and control’. Each phrase is within HAL and is available to be edited by the user, appended and then randomly chosen by the programme and then finished with the accurate placement of the numerical effect estimate, details of the number of studies contributing to that estimate and the total number of relevant participants.
  - Acknowledgements: seeded with a standard sentence which highlights how the review has been written with the help of RevManHAL and a hyperlink to the programme’s webpage.
2. The possibility of creating and managing language files. This allows reviewers to insert text in their preferred language. Current languages for which files already exist are English, Spanish, German and Mandarin. It does not translate but generates a first draft in one of the chosen languages and also gives users opportunity to create additional files for more languages.

HAL uses a repository of phrases to construct sentences currently in four already constructed language files (English, German, Spanish, Mandarin) supplied with the programme. These files follow an XML structure that RevMan supports. As with any other functionality offered by HAL, reviewers can edit these language files and add or remove as many options for each statement as they want. Moreover, HAL contains a language file creation interface to allow new language files to be made. HAL has, therefore, the capacity to write and be used in reviews in any language that reads left to right.

## Strengths

RevManHAL creates clear and readable text which is instantly added to the review. RevManHAL is already saving reviewers an extraordinary amount of time. Overall, it helps researchers avoid the fatigue and lack of accuracy that the necessary repetitive tasks entail. With the auto-generation of text, avoidance of needless repetition of effort, RevManHAL and its successors should allow authors to be able to produce and maintain reviewers faster. Even now, as a consequence, reviews reach patients, carers and interested parties faster.

RevManHAL also adds highlighted markers, which show the reader where the HAL-generated text starts and ends. These markers can be deleted by the author once everything has been checked.

## Limitations

A clear limitation is that HAL is tied to the use of RevMan and Cochrane reviews are a minority of systematic reviews overall [15–17]. However, many, outside of the Cochrane Collaboration, still use the RevMan software and export the product to format for other types of dissemination. HAL does not relate to any other programmes. Certainly linking to DistillerSR [18] or Covidence [19] could be of great value and move HAL outside of its RevMan constraints. HAL remains a proof-of-concept prototype. It has bugs (e.g. data where risk estimate is impossible to estimate generates nonsense text) and is limited. However, it shows how auto-generation of readable multilingual text for these reviews is possible.

## The future

HAL’s template output is constrained to meet the needs of Cochrane reviews. This does not have to be that way. With a structured dataset—such as what RevMan creates—output could be anywhere for any format, for any language.

**Fig. 2:**
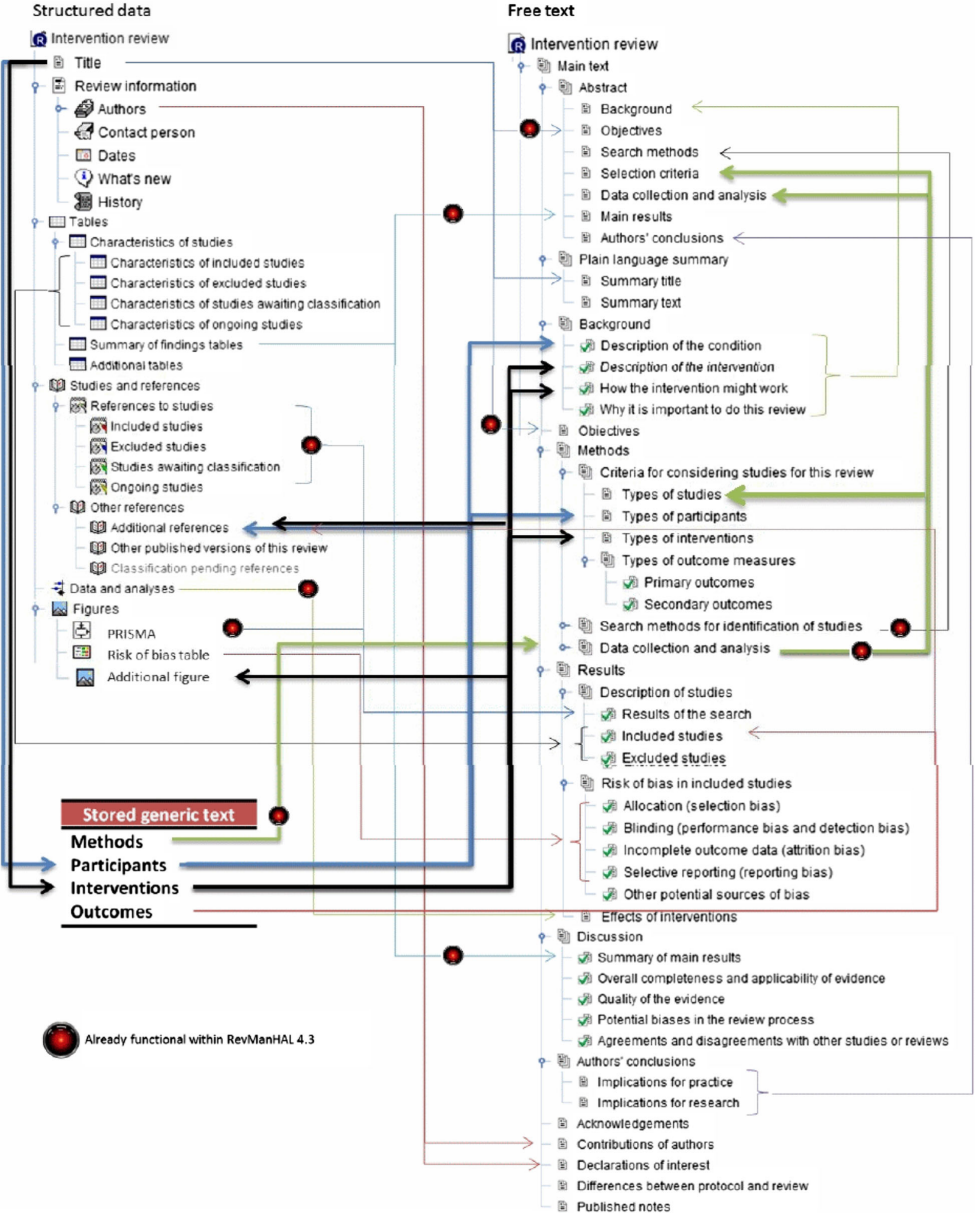
Current and potential data linkage within RevMan

The current RevManHAL v.4 compiles a series of basic functions aimed to help Cochrane reviewers produce reviews faster and more accurately. However, there are many more functions possible with greater or lesser amounts of programming. The structure of a systematic review lends itself to auto-generation of text in many other sections (Fig. 2) such as the description of the included/excluded studies. Some of the main functionality that we hope to add in next versions include:

1. Editing
  - Simple
    As with any sophisticated word-processing software, auto-editing should be able to be programmed in. For example, ‘data is’ should be auto changed to ‘data are’, ‘teh’ to ‘the’. The structure of the review, however, allows certain functions to be carried out only in certain pre-stated areas. For example, if all statements in tables are to be terminated by a ‘.’, then this action can be pre-specified to be undertaken only in this area [20]. This HAL plug-in software is currently being tested.
  - Complex
    For electronic publication review, protocols often shift tense. Protocols are written in the future tense, predicting actions (‘we will……’), and the reviews in past (‘we found…..’), often with conditional clauses (‘should we have found this we would have……..’). Experimentation with programming using an appropriate ‘phrase bank’ has shown that auto-shifting of tense is possible and reliable [20].
2. Using prepared repositories of descriptions of interventions and illnesses
  Should data inputted by reviewers be structured—as forced by the RevMan programme—more reliable links can be made. For example, the title could link to repositories of text regarding interventions, drugs and illnesses. Such a repository would be created and maintained, with the option to be downloaded and edited by the authors. For example, titles are often structured ‘Intervention X for condition Y’—such as ‘Aspirin for headache’. The appearance of the word ‘aspirin’ could link to data banks of drug names, descriptions and diagrams. Moreover, HAL could link ‘headache’ to already well-written open-access text on this problem. This same function is possible from the descriptions of outcomes reported within the review. Should an outcome be derived from, for example, a well-known measurement scale, such as a named measure of pain, the name could trigger auto-seeding of already written, publically accessible, text describing and referencing the properties and meaning of the scale.
3. Using data within the review to generate new text
  As data within the characteristics of included studies tables are already often highly structured, these lend themselves to further summation. In every review, it is necessary to provide an overview of the nature and conduct of the included studies. It would not be problematic to produce text around averages produced by machine. For example, for the description of participants, the auto-generated text could read ‘The average size of trial was 45 people (SD 35, median 22.4, mode 22) and around 45% were men (limitations of reporting preclude fully accurate percentages)’—where the numbers are calculated by machine and the text is added from a phrase repository. If data are structured, as is so often required in such tables, text could go on to describe the diagnoses of participants and their history. Further text could be auto-generated in just the same way to help overview methods, interventions and outcomes.
4. Developing outputs of text for dissemination in other ways
  - Languages
    Currently, RevManHAL has the potential to produce text for limited but essential parts of the review in any left-to-right reading language. It would seem important to expand this scope to more parts of the review.
  - Plain language
    RevManHAL currently writes auto-generated text in a form based on a scientific writing style. Numbers are prominent, but some readers may find these off-putting. It is entirely a matter of programming to allow informative plain-language summaries to be produced by machine in multiple languages/dialects.
  - Other web formats
    Wikipedia is, and is likely to remain, a frequently used source of information [21]. Its use is ubiquitous and entirely free to many low- and middle-income countries—not even incurring download charges to many parts of Africa or the Middle East [22]. Referenced evidence tables can be readily written and outputted from a programme such as RevManHAL in formats that are fully compatible for pasting into Wikipedia pages. Early experiments have, thus far, been successful [23].
  - Social media
    There is evidence that dissemination though social media increases at least ‘product placement’ of evidence [24]. All subsidiary products from a large review take some time to create, and consideration and skill to compile—even the short 140 character phrases of Twitter or Weibo. With all data within a repository such as an XML file, it is not difficult for a machine to take from a bank of phrases a set of short statements appropriate to the target review data which convey some sense of the findings and their implications with a link to the full review online. As with all machine-generated text, these would be presented to the authors of the review for editing.

## Conclusion

For every drug, device, care package, and therapy, there are the potential for many comparisons generating impossible numbers of reviews and overviews [25]. In turn, these reviews will swiftly become outdated in the light of new evidence. Finally, too much of the evidence is produced in a form that is inaccessible to most. There is the dual barrier of sole use of the English language combined with a scientific writing style that takes reviewers a long time to create.

Collective invention is a concept familiar to economists and historians [26]. When the time is right, collective efforts can result in great swift steps forward as was seen in the genesis of the Industrial Revolution in Britain from the 1750s. The last two decades have seen the process of systematic reviewing of healthcare go from cottage industry to attempting to meet industrial demands for health evidence. In the next years, software and data repositories will take a leap forward. With more journal articles containing structured datasets and the availability of study data repositories, there may be less reliance on the highly manual process of data entry into programmes such as RevMan. In the meantime, however, RevManHAL can help shorten the time taken from finalising data entry to finishing write-up.

## Abbreviations

DistillerSR: A specific tool for assisting systematic reviews
GRADEpro: A specific guideline development tool
PRISMA: Preferred Reporting Items for Systematic Reviews and Meta-Analyses
RevMan: Review Manager programme
RevManHAL: Review Manager programme add-on described in the paper (no acronym)
XML: Extensible Markup Language
